# Editorial: Microbial Secondary Metabolites: Recent Developments and Technological Challenges

**DOI:** 10.3389/fmicb.2019.00914

**Published:** 2019-04-26

**Authors:** Bhim Pratap Singh, Mostafa E. Rateb, Susana Rodriguez-Couto, Maria de Lourdes Teixeira de Moraes Polizeli, Wen-Jun Li

**Affiliations:** ^1^Department of Biotechnology, Aizawl, Mizoram University, Aizawl, India; ^2^School of Computing, Engineering and Physical Sciences, University of west of Scotland, Paisley, United Kingdom; ^3^IKERBASQUE, Basque Foundation for Science, Bilbao, Spain; ^4^Department of Biology, Faculty of Philosophy, Science and Letters of Ribeirão Preto, University of São Paulo, São Paulo, Brazil; ^5^State Key Laboratory of Biocontrol and Guangdong Provincial Key Laboratory of Plant Resources, School of Life Sciences, Sun Yat-Sen University, Guangzhou, China

**Keywords:** microbial, secondary, metabolite, change, challenges

## Introduction

Microbial secondary metabolites, like antibiotics, pigments, growth hormones, antitumor agents, and others, are not essential for the growth and development of microorganism, but they have shown a great potential for human and animal health (Ruiz et al., 2010). Among the microorganisms producing the above-mentioned compounds, bacteria, including actinobacteria, and fungi produce a diverse array of bioactive small molecules with significant potential to be used in medicine (O‘Brien and Wright, 2011). These bioactive compounds are mainly produced by the activation of cryptic gene clusters which are not active under normal conditions and, thus, the expression of these clusters would be helpful in the exploitation of the chemical diversity of microorganisms (Pettit, 2011; Xu et al., 2019).

Although several reports on microbial secondary metabolites have been published in recent years (Passari et al., 2017; Zothanpuia et al., 2018; Overy et al., 2019), our understanding to enhance the production of bioactive secondary metabolites is still limited. The research topic “Microbial Secondary Metabolites: Recent Developments and Technological Challenges” comprises 25 articles covering important aspects on biodiversity, exploitation and utilization of microbial resources (terrestrial, marine, and endophytic) for the production of secondary metabolites together with their biological functions.

The current knowledge and potential of marine fungi for producing anticancer compounds has been reviewed (Deshmukh et al.) and the ability of the sea-derived *Streptomyces helimycini* for the production of actinomycins is presented (Zhu et al.). In a very interesting study, Wakefield et al. proved that the co-cultivation of fungi and bacteria led to the production of new secondary metabolites. There is a growing interest in looking for unique sources for the exploration of novel microbial populations having prospective to produce bioactive natural products. Thereby, the bacterial and fungal population obtained from *Aquilaria malaccensis* tree and soil enhanced the production of agarospirol within 3 months of artificial infection (Chhipa and Kaushik).

The present research topic includes four important research papers dealing with the production of bioactive secondary metabolites. Thus, a study by Alenezi et al. emphasized that the biological activity of *Aneurinibacillus migulans* isolates was directly correlated with the production of a new gramicidin. Narsing Rao et al. has focused on the importance of pigments originated from fungi and bacteria and their wide applications in health and industry. The article by Li et al. presented the production of somalimycin, a new antimycin-type depsipeptide, from a mutant of the deep-sea-derived *Streptomyces somaliensis*. Similarly, Thøgersen et al. demonstrated the production of the potentially antibacterial compounds violacein and indolmycin by a *mae*A mutant of the sea bacterium *Pseudoalteromonas luteoviolacea*.

A cluster of three articles gives emphasis to the biosynthetic gene clusters involved in microorganisms for the production of secondary metabolites. Hence, Derntl et al. demonstrated the role of genes, namely *sor*1, *sor*3, and *sor*4 of the orbicillinoid gene cluster and disclosed the function of *sor*4 which was not known. Another article by Rojas-Aedo et al. explained the role of the *adr* gene cluster involved in the biosynthesis of the potent antitumor compoundandrastin A in *Penicillium roqueforti*. In this article, the authors also have demonstrated that all the 10 genes of *adr* gene cluster were essential for the production of andrastin A. Lastly, Nah et al. reviewed the potential of the phylum Actinomycetes for natural production (NP) through biosynthetic gene clusters (BGC) heterologous expression systems as well as recent strategies specialized for the large-sized NP BGCs in *Streptomyces* heterologous hosts.

Other important candidates for the production of secondary metabolites are the endophytic microorganisms which were addressed by Mefteh et al. Thus, they presented that plants under biotic stress offered new and unique endophytes with diverse bioactivities as compared to healthy plants. Sharma et al. reported that the application of dietary components like grape skin and turmeric extracts enhanced the production of cryptic and bioactive metabolites, with anti-oxidant and antibacterial potential, by the endophytic fungus *Colletotrichum gloeosporioides*. Also, the endophytic fungi *Chaetomium globosum* isolated from Egyptian medicinal plants, proved to have anti-rheumatoid activity (Abdel-Azeem et al.).

In summary, the articles gathered in the research topic “Microbial Secondary Metabolites: recent development and Technological Challenges” explore the role of microorganisms from different sources showing biological activities. This will further enhance the present knowledge on the potential of microbial secondary metabolites in health and industry. One challenge which needs to be answered is the development of methods to understand the detailed mechanisms of cryptic genes and their relation to the production of bioactive compounds. Researchers also need to give more emphasis on the co-cultivation of different microorganisms having positive synergistic effect to produce novel bioactive molecules. We believe that this special issue gives some in-depth information about one of the important matters of the microbial world. Finally, our great thanks to all contributions, in total 165 authors, for the cohesive information in the form of reviews and research articles which have been compiled in this ebook. We strongly believe that the information compiled and presented in this ebook will be useful for the readers and will be the basis for the future investigation on “microbial secondary metabolites.”

## Author Contributions

All authors mentioned have made significant contributions in the production of the editorial and have approved it for publication.

### Conflict of Interest Statement

The authors declare that the research was conducted in the absence of any commercial or financial relationships that could be construed as a potential conflict of interest.
